# Geldanamycins: Potent Hsp90 Inhibitors with Significant Potential in Cancer Therapy

**DOI:** 10.3390/ijms252011293

**Published:** 2024-10-20

**Authors:** Omeima Abdullah, Ziad Omran

**Affiliations:** 1 College of Pharmacy, Umm Al-Qura University, Makkah 21955, Saudi Arabia; oaabdullah@uqu.edu.sa; 2 King Abdullah International Medical Research Center, King Saud Bin Abdelaziz University for Health Sciences, Jeddah 21423, Saudi Arabia

**Keywords:** geldanamycin, ansamycin, cancer, Hsp90, mutasynthesis, PROTAC

## Abstract

Geldanamycin, an *ansa*-macrolide composed of a rigid benzoquinone ring and an aliphatic *ansa*-bridge, was isolated from *Streptomyces hygroscopicus*. Geldanamycin is a potent heat shock protein inhibitor with remarkable antiproliferative activity. However, it shows pronounced hepatotoxicity in animal models and unfavorable pharmacokinetic properties. Four geldanamycin analogs have progressed through various phases of clinical trials, but none have yet completed clinical evaluation or received FDA approval. To enhance the efficacy of these Hsp90 inhibitors, strategies such as prodrug approaches or nanocarrier delivery systems could be employed to minimize systemic and organ toxicity. Furthermore, exploring new drug combinations may help overcome resistance, potentially improving therapeutic outcomes. This review discusses the mechanism of action of geldanamycin, its pharmacokinetic properties, and the various approaches employed to alleviate its toxicity and maximize its clinical efficacy. The main focus is on those derivatives that have progressed to clinical trials or that have shown important in vivo activity in preclinical models.

## 1. Introduction

Despite the considerable progress made in human genomics and drug discovery in the twenty-first century, cancer remains a leading cause of death worldwide, accounting for at least 10 million deaths in 2020 [1]. Cancer is linked to the overexpression of proteins or chaperones associated with protein folding [2], including heat shock proteins (Hsps), the first identified chaperones, named for their increased levels when cells are exposed to higher-than-normal temperatures. The 90 kDa Hsp (Hsp90), the most abundant human Hsp, plays a crucial role in cellular proteostasis by controlling the folding, stabilization, activation, and degradation of more than 400 client proteins [3]. Among these client proteins are many that are considered oncogenic, such as hypoxia-inducible factor-1 (HIF-1), platelet-derived growth factor receptor (PDGFR), the estrogen receptor (ER), and human epidermal growth factor receptor 2 (HER2) [4]. Thus, inhibiting Hsp90 represents an attractive strategy for cancer management.

**Figure 1:**
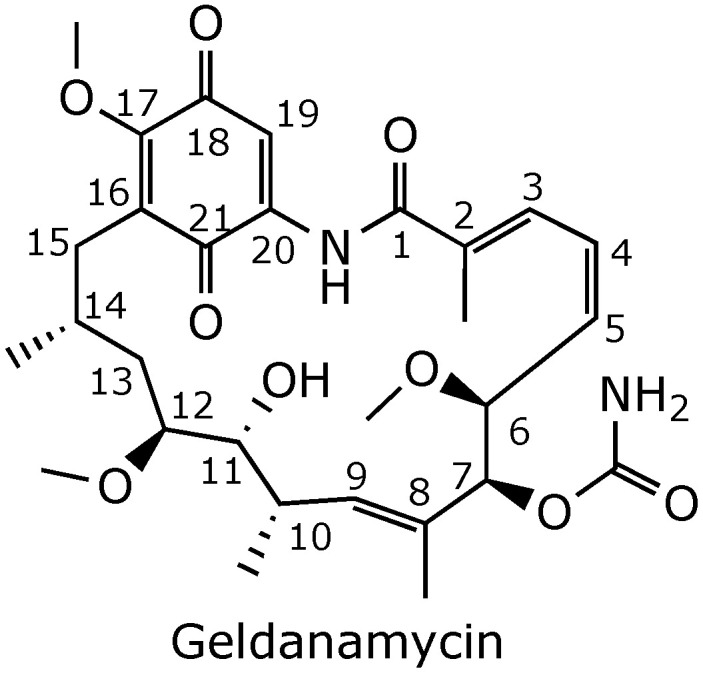
Chemical structure of geldanamycin [5].

One potent Hsp90 inhibitor is geldanamycin (Figure 1), a compound first isolated from culture filtrates of the soil actinomycete *Streptomyces hygroscopicus* var. *geldanus* var. *nova* by C. DeBoer and his colleagues in 1970 [5]. Geldanamycin was initially shown to have moderate activity against protozoa, bacteria, and fungi [5], but it was later found to possess potent anticancer activity against a wide range of human cancers through its inhibition of Hsp90 [6]. However, despite its promising anti-cancer potential in vitro, geldanamycin was not pursued for clinical use because of its hepatotoxicity in animal models and unfavorable pharmacokinetic properties [7]. Nevertheless, over the past three decades, numerous analogs of geldanamycin have been developed in an effort to overcome these limitations, and several of these derivatives have progressed through different phases of clinical trials [8]. In this review, we will discuss the mechanism of action of geldanamycin and the different approaches taken to alleviate its toxicity and improve its pharmacokinetics.

## 2. Heat Shock Proteins

Hsps are induced under cellular stress situations, such as excessive heat, UV radiation, nutrient deprivation, or oxygen deficiency [9,10], and they are essential for cell survival. Hsp90 is a highly conserved housekeeping gene found in both prokaryotes and eukaryotes [11,12]. In human cancer patients, its overexpression, along with that of heat shock factor-1 (HSF-1), a transcriptional regulator of many Hsps, is linked to poor prognosis, tumor invasion, metastasis, and treatment resistance [13,14,15,16]. In mammalian cells, Hsp90 has four functional paralogs: Hsp90α and Hsp90β, localized in the cytosol and nucleus; tumor necrosis factor receptor-associated protein 1 (TRAP1), localized in the mitochondria; and HspB1, localized in the endoplasmic reticulum [17,18,19]. Structurally, the cytosolic Hsp90α is composed of 855 amino acids and has a molecular weight of 98.1 kDa. Hsp90 exists as a homodimer, with each monomer consisting of four structural domains: an *N*-terminal nucleotide-binding domain (NTD) that has ATPase activity and is linked through an unstructured flexible linker region to a client protein-binding middle domain (MD), followed by a *C*-terminal domain (CTD) involved in dimerization [12,20,21,22,23,24,25].

Hsp90 mitigates cellular stress by stabilizing a diverse range of more than 400 client proteins, many of which are considered oncogenic. Hsp90 client proteins can be broadly categorized into the following groups [16,26]:

(a) Protein kinases, such as epidermal growth factor receptor (EGFR), human epidermal growth factor receptor 2 (HER2), cyclin-dependent kinase 4 (CDK4), and rapidly accelerated fibrosarcoma 1 (RAF1).
(b) Transcription factors, such as p53, HSF-1, and HIF-1.
(c) Steroid hormone receptors.
(d) Chromatin remodeling factors.
(e) Telomerase.

In the nucleosome, Hsp90 interferes with chromatin remodeling and telomerase maintenance [23]. In the cytosol, it facilitates the folding and maturation of nascent polypeptides into functional three-dimensional proteins [27,28]. Additionally, it refolds proteins that have been denatured due to cellular stress. This unique ability to fold both nascent and denatured proteins, including mutant forms, is an important contributor to carcinogenesis, making Hsp90 an ideal target for drug development [9,29,30], as molecules that can inhibit Hsp90 by binding to its different sites hold potential as anticancer drugs [30]. Three major approaches are currently being pursued to inhibit Hsp90 function:

(a) Targeting the NTD to inhibit Hsp90 ATPase activity (e.g., geldanamycin and its analogs [31]).
(b) Targeting CTD to change Hsp90 conformation (e.g., novobiocin [32,33]).
(c) Inhibition of co-chaperone binding, such as Cdc37 (e.g., gedunin [34,35,36] and celastrol [37,38]).

## 3. Mode of Action of Geldanamycin

**Figure 2:**
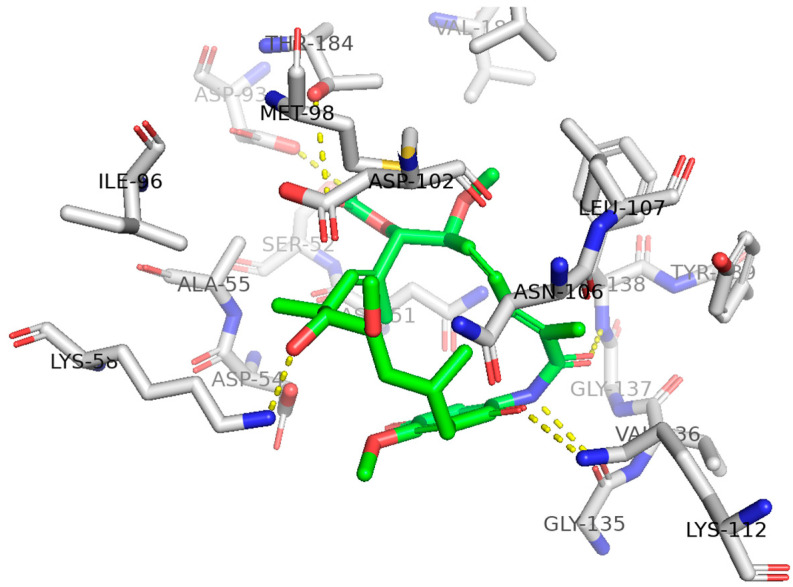
Binding interactions of geldanamycin with Hsp90. The structure was generated from PDB: 1YET [31].

**Figure 3:**
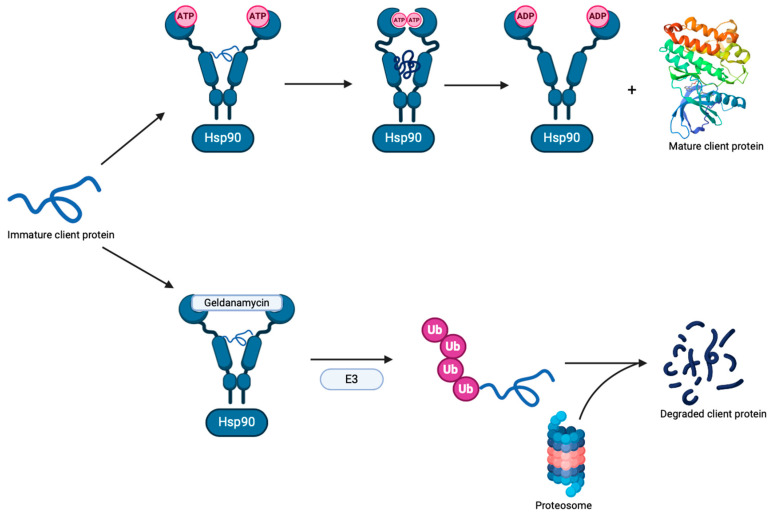
Inhibition of Hsp90 by geldanamycin and subsequent degradation of its client proteins [41].

Geldanamycin binds to the *N*-terminal ATP binding site of Hsp90 in a U-shaped conformation, in which the benzoquinone ring and the aliphatic chain adopt a nearly parallel orientation, and the lactam group adopts a *cis* configuration [31,39] (Figure 2). Geldanamycin interacts with the Hsp90 NTD through a network of seven hydrogen bonds with N51, K58, D93, I96, G97, N106, and G135. Geldanamycin further interacts through van der Waals interactions with L48, S52, G95, D102, V136, G137, T152, G183, T184, and V186 inside the NTD [40]. Upon binding with geldanamycin, Hsp90 recruits the carboxy-terminus of the HSP70-interacting protein, an E3 ubiquitin ligase. The client proteins are then ubiquitylated and further degraded by the 26S proteasome [41] (Figure 3).

In addition to inhibiting Hsp90, geldanamycin and its benzoquinone analogs have been shown to bind to the mitochondrial membrane voltage-dependent anion channel (VDAC) through hydrophobic interactions. This binding increases intracellular Ca^2+^ levels and reduces the plasma membrane cationic current, leading to the inhibition of urokinase activity and the suppression of cell invasion. Interestingly, geldanamycin derivatives lacking the benzoquinone ring do not affect the cationic current and have little effect on the intercellular Ca^2+^ concentration [8,42].

## 4. Geldanamycin Structure, Biosynthesis, and Toxicity

**Figure 4:**
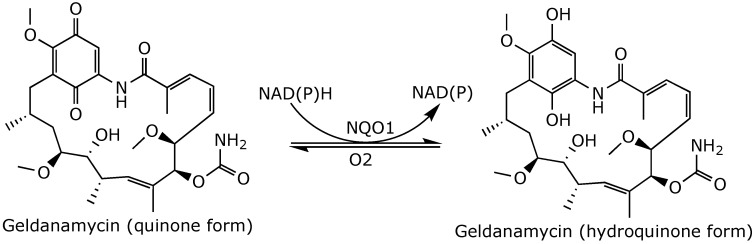
Two-electron reduction of geldanamycin by NQO1 [44].

Geldanamycin is an *ansa*-macrolactam with two distinctive structural features: a rigid benzoquinone ring and an aliphatic *ansa*-bridge. These features are interconnected to create a characteristic basket-like (*ansa*) structure [43]. Geldanamycin is metabolized via a two-electron reduction of its benzoquinone ring to form a hydroquinone by NAD(P)H: quinone oxidoreductase 1 (NQO1) (Figure 4) [44]. The hydroquinone form of geldanamycin is more active and has a higher affinity for Hsp90 than the quinone form, as it forms two additional hydrogen bonds with the D54 and D93 residues in the Hsp90 NTD [45].

**Figure 5:**
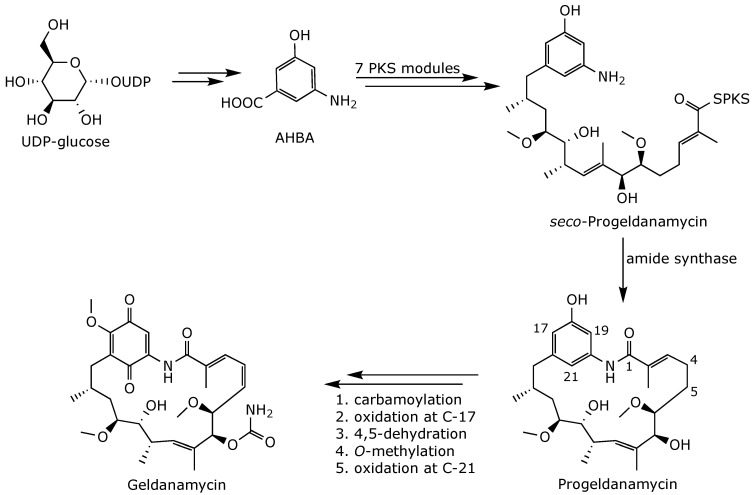
Biosynthesis of geldanamycin [51,52].

The biosynthetic machinery used by *S*. *hygroscopicus* to produce geldanamycin consists of seven polyketide synthase (PKS) modules and an additional post-PKS enzyme set, all of which are encoded by the *gdm* biosynthetic gene cluster [46]. Geldanamycin biosynthesis begins with 3-amino-5-hydroxybenzoic acid (AHBA), which is produced from *D*-glucose [47] (Figure 5). AHBA is loaded onto the starter module and undergoes chain extension by the seven PKS modules using one malonate, four methylmalonates, and two methoxymalonate extender units to yield *seco*-progeldanamycin. This intermediate is subsequently cyclized into progeldanamycin by an amide synthase and then released from PKS. The progeldanamycin is then further transformed into geldanamycin by a series of reactions, including amidination, several oxidations, *O*-methylation, and a C4–C5 desaturation reaction, performed by a set of tailoring enzymes [48,49,50,51].

**Figure 6:**
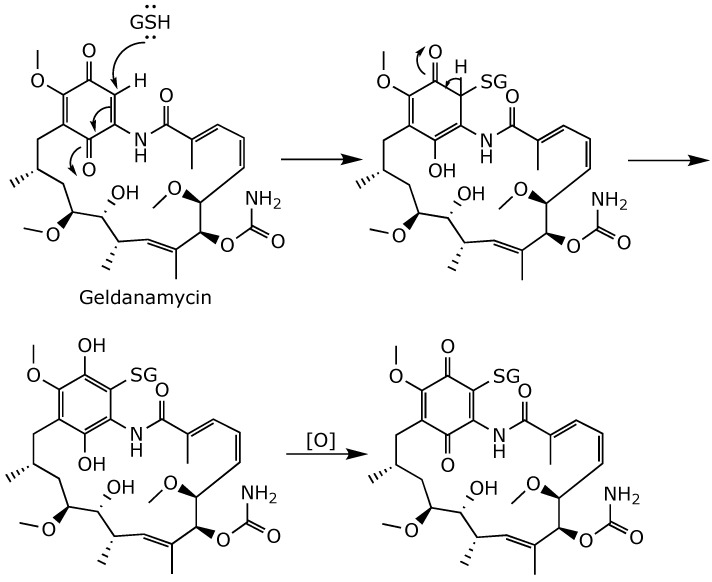
1,4-Michael reaction cascade between geldanamycin and GSH [54].

The resulting product has high hydrophobicity, which is responsible for its low water solubility and limited oral bioavailability [8]. On the other hand, the pronounced hepatotoxicity of geldanamycin is attributed to the Michael acceptor feature of its benzoquinone ring, as a metabolic Michael-type addition of glutathione (GSH) at position C(19) limits the ability of mammalian cells to neutralize reactive oxygen species (Figure 6) [44]. Interactions with thiol groups in crucial cellular proteins are also suggested to play a significant role in the geldanamycin toxicity mechanism [53].

**Figure 7:**
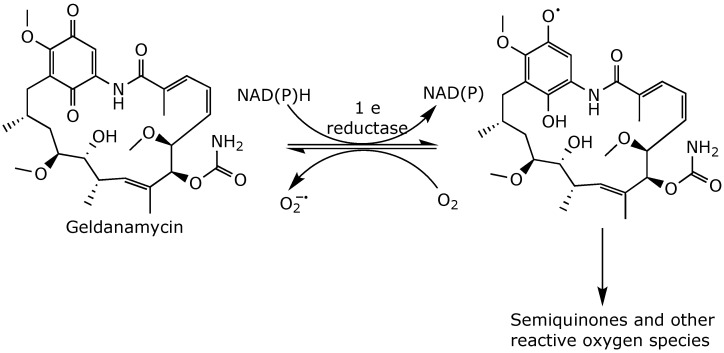
One-electron reduction of geldanamycin [44].

Additionally, the quinone moiety can be metabolized by one-electron reductases generating unstable semiquinones and superoxide radicals [55] (Figure 7). These superoxides can subsequently generate reactive oxygen species that can harm cells by damaging essential macromolecules [56]. Given that the liver contains high levels of one-electron reductases [57], the one-electron metabolism of quinones contributes to geldanamycin’s hepatotoxicity [44].

Numerous efforts have been made to enhance the pharmacodynamic and pharmacokinetic properties of geldanamycin through structural modifications of both the benzoquinone core and the *ansa*-chain through total synthesis, semisynthesis, or mutasynthesis. Generally, modifying the *ansa*-bridge reduces anticancer activity [54,58], likely because of the restricted flexibility of the *ansa*-bridge, which is crucial for binding with Hsp90. Therefore, this review focuses on alterations of the highly amenable benzoquinone ring, either by substituting position C(19) or C(17) or by substituting the ring with aromatic or heteroaromatic rings.

## 5. Substitution of Position C(19)

**Figure 8:**
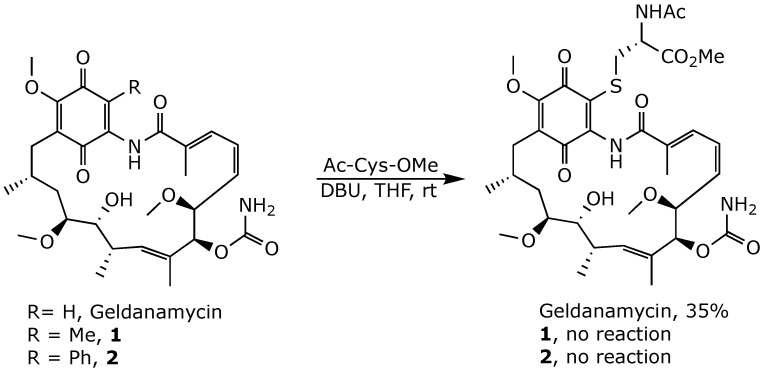
Addition of *N*-acetylcysteine methyl ester to geldanamycin and its derivatives substituted at position 19 [59,60,61].

Blocking position 19 on the benzoquinone ring was proposed to suppress the conjugation of glutathione and other nucleophiles. Consequently, Moody et al. developed a series of geldanamycin derivatives with alkyl, aryl, or heteroaryl substituents introduced at position 19 [59,60,61]. Indeed, in contrast to geldanamycin, compounds **1** and **2** did not undergo Michael addition when treated with *N*-acetylcysteine methyl ester (Figure 8) [59].

**Figure 9:**
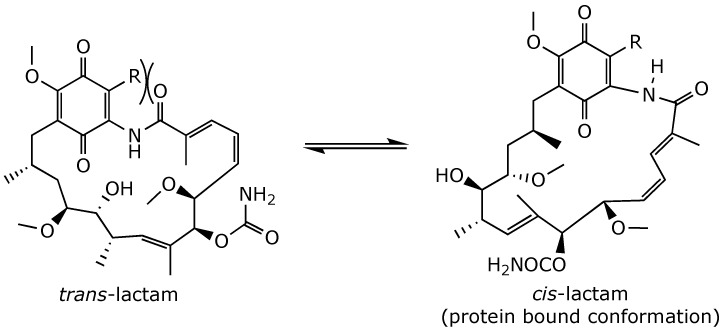
*Trans–cis*-lactam isomerization in geldanamycin [45].

Interestingly, the 19-substituted geldanamycin derivatives were significantly more polar than the parent geldanamycin. More importantly, position 19 substituents induced a favorable conformational switch of the *trans*-lactam group into the *cis*-form, which is required for binding to the *N*-terminal ATP site (Figure 9). Consequently, these compounds showed potent inhibition of Hsp90 and remarkably reduced its toxicity up to 400-fold, compared to the parent unsubstituted compound when tested in human umbilical vein endothelial cells (HUVECs). Furthermore, 19-substituted geldanamycin analogs exhibited reduced toxicity in mouse hepatocytes. However, the growth-inhibitory potency of these derivatives was lower compared to their parent 19-unsubstituted quinones [45].

## 6. Substitution of Position C(17)

**Figure 10:**
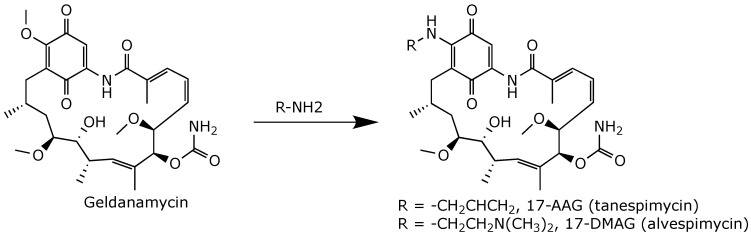
Semi-synthesis of 17-AAG and 17-DMAG [62,63].

Substitution of the methoxy group at position 17 by a stronger electron-donating group (i.e., an amine) reduced the electrophilicity of the benzoquinone ring and increased the water solubility of geldanamycin. This reaction yielded the amino derivatives 17-allylamino-demethoxygeldanamycin (17-AAG, tanespimycin) and 17-dimethylaminoethylamino-17-demethoxygeldanamycin (17-DMAG, alvespimycin) from geldanamycin by semi-synthesis (Figure 10).

The derivative 17-AAG retained the potent anticancer activity of geldanamycin but with reduced hepatotoxicity and improved bioavailability. It was the first derivative of geldanamycin to enter clinical trials for a wide variety of human cancers [64,65]. To date, 17-AAG has been administered either alone or in combination with various drugs in a total of 38 phase 1 and phase 2 clinical trials [16]. The derivative 17-AAG was also advanced to an NIH-funded phase 3 clinical trial for relapsed–refractory multiple myeloma [65]. Unfortunately, although 17-AAG showed therapeutic benefits, its clinical development was halted due to its low water solubility and its hepatotoxicity.

By contrast, 17-DMAG offers several advantages over 17-AAG, such as higher water solubility, better bioavailability, reduced metabolism, and greater anticancer activity [66]. It can be administered orally or intravenously. The derivative 17-DMAG has been used in seven clinical trials as a monotherapy or in combination with other agents against various solid and hematological tumors [16]. Although 17-DMAG showed promising results against myelogenous leukemia [67] and HER2+ metastatic breast cancer [68], its clinical development was stopped due to its higher toxicity compared to 17-AAG [69].

**Figure 11:**
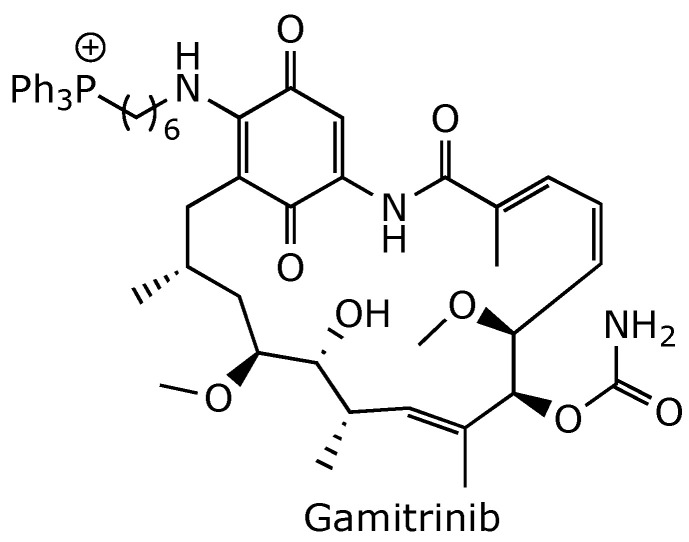
Chemical structure of gamitrinib, a mitochondria-targeted HSP90 inhibitor [70].

The toxicity displayed by geldanamycin and its analogs 17-AGG and 17-DAMG was addressed by the development of a class of hybrid molecules, exemplified by gamitrinib (Figure 11). These molecules were obtained by linking geldanamycin to an efficient mitochondrial import carrier, such as triphenyl phosphonium. Gamitrinib has been shown to inhibit the activity of the Hsp90 paralog TRAP1, which is specifically localized in the mitochondrial matrix. Gamitrinib induces mitochondrial dysfunction, loss of membrane potential, and membrane rupture, leading to rapid tumor cell death. In contrast to geldanamycin and its 17-AGG and 17-DAMG analogs, gamitrinib showed no toxicity to normal cells or tissues and did not disrupt Hsp90 homeostasis in cellular compartments outside the mitochondria [70]. Gamitrinib was advanced to phase 1 clinical trials to treat patients with advanced cancer [71], but the trial was suspended in 2023 because of the limited drug supply [16].

**Figure 12:**
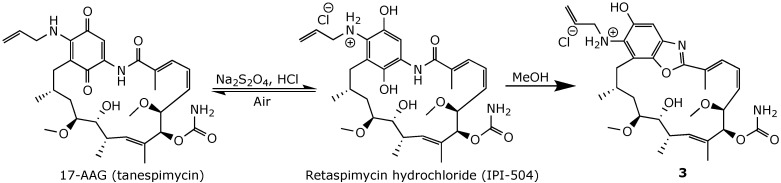
Chemical synthesis of retaspimycin hydrochloride and its locked benzoxazole analog **3** [72].

Another approach employed to address the drawbacks of 17-AAg was the development of retaspimycin (IPI-504), a highly water-soluble hydroquinone hydrochloride derivative of 17-AAG (Figure 12) [72]. Once in the systemic circulation, retaspimycin hydrochloride is deprotonated and converted into free base retaspimycin, which is subsequently oxidized to 17-AAG. Retaspimycin hydrochloride was evaluated in clinical trials for non-small cell lung cancer (NSCLC) [16] and for ERBB2-positive breast cancer [73], but it failed to meet the criteria for study expansion. A phase III RING trial of retaspimycin hydrochloride for refractory gastrointestinal stromal tumors was also terminated due to high mortality [16].

**Figure 13:**
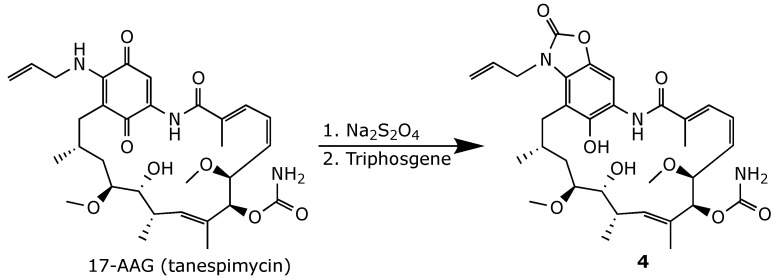
Chemical synthesis of geldanamycin carbamate derivative **4** [72].

**Figure 14:**
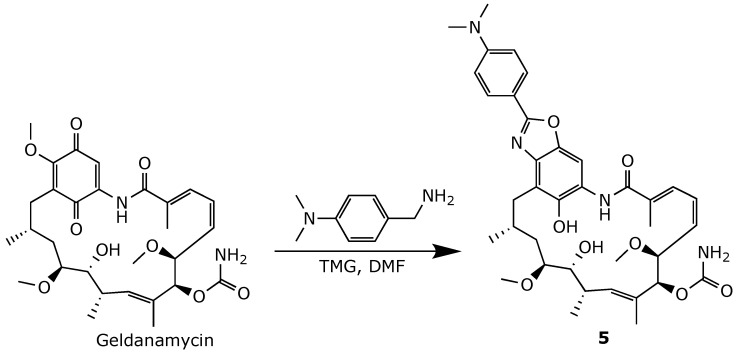
Chemical synthesis of geldanamycin benzoxazole derivative **5** [74].

The oxidation of retaspimycin back into 17-AAG was prevented by developing locked benzoxazole **3** and hydroquinone carbamate **4** (Figure 12 and Figure 13). However, both compounds exhibited a significantly reduced affinity to Hsp90, likely due to the loss of crucial H-bonding with the binding site [72]. Conversely, phenylbenzoxazole derivative **5**, obtained by treating geldanamycin with dimethylaminobenzylamine in the presence of a strong organic base, such as tetramethylguanidine (TMG) (Figure 14), retained an in vitro anticancer activity comparable to that of geldanamycin, with slightly improved selectivity [74].

**Figure 15:**
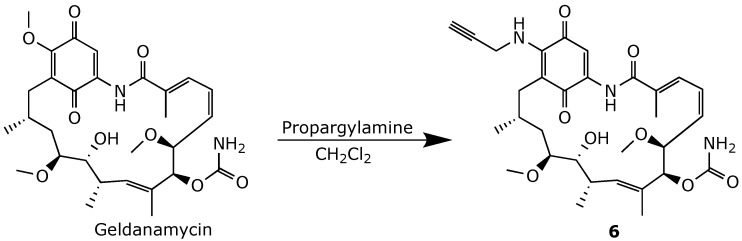
Chemical synthesis of 17-propargylamine-17-demethoxygeldanamycin **6** [75].

The propargyl analog of 17-AAG, 17-propargylamine-17-demethoxygeldanamycin (compound **6**), was synthesized and evaluated by Shen and his coworkers [75]. Like 17-AAG and 17-DMAG, compound 6 was obtained by reacting geldanamycin with propargylamine (Figure 15). Alkyne derivative **6** exhibited potent in vitro antiproliferative activity, particularly against the breast cancer cell line MDA-MB-231, with an IC50 of 60 nM. An in vivo hepatotoxicity evaluation of compound **6** demonstrated that it induced lower levels of liver toxicity than geldanamycin and 17-AAG, as evidenced by reduced levels of aspartate aminotransferase and alanine aminotransferase. Furthermore, compound **6** displayed more potent antitumor activity in an MDA-MB-231 xenograft model, compared to 17-AAG.

**Figure 16:**
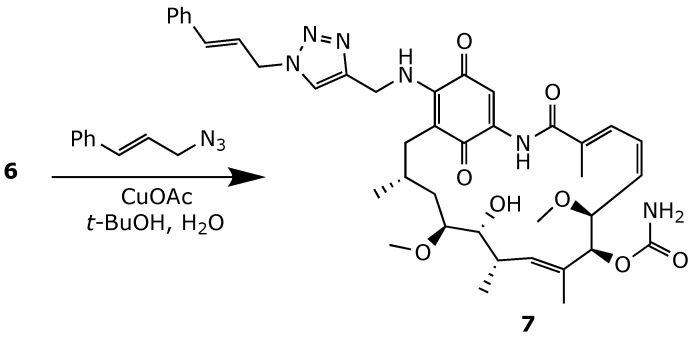
Synthesis of the triazole derivative **7** [76].

Further attempts to address the limitations of 17-AAG recently led Skrzypczak et al. to develop a series of triazole analogs using click chemistry, starting from the alkyne derivative **6** (Figure 16). Among the 35 diverse compounds synthesized, compound **7** showed improved binding affinity to Hsp90 and enhanced apoptotic activity in SKBR-3 and SKOV-3 cancer cell lines when compared to geldanamycin. Compound **7** also displayed a 3-fold decrease in in vitro toxicity in the healthy HDF cell line [76].

**Figure 17:**
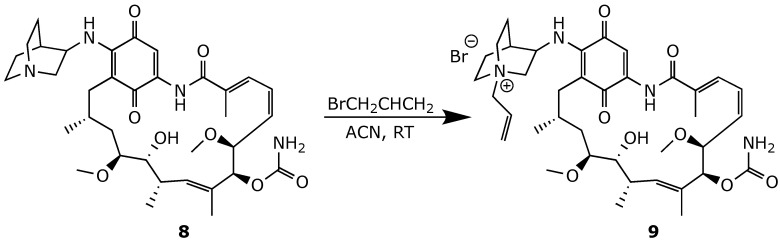
Quaternization of the quinuclidine analog of geldanamycin **8** [43].

Similar results were obtained when replacing the C(17)-OMe group of geldanamycin with quinuclidine, a rigid, bulky amine motif, to generate compound **8** (Figure 17). However, attempts to increase the water solubility of this derivative by forming quaternary ammonium salts resulted in reduced cytotoxicity against both cancerous and healthy cell lines [43].

## 7. Replacement of the Benzoquinone Ring

**Figure 18:**
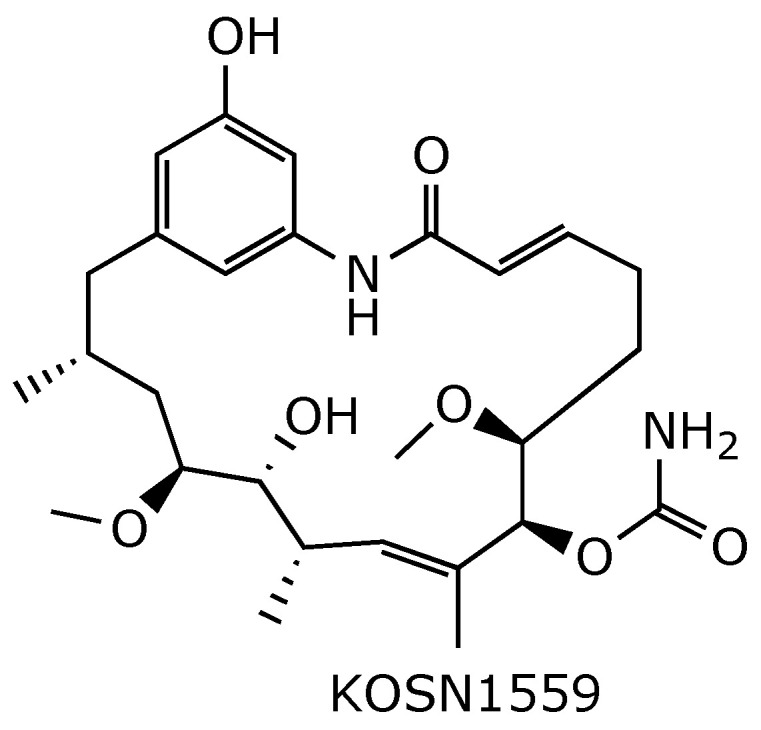
Chemical structure of KOSN1559 prepared by engineered biosynthesis [77].

Since the benzoquinone core is responsible, at least in part, for the hepatotoxic side effects of geldanamycin, many efforts have focused on replacing benzoquinone with other rings lacking redox-sensitive quinone or hydroquinone moieties. This has been made possible through genetic engineering. For example, the phenolic derivative KOSN1559 [77] (Figure 18) was obtained through gene manipulation of the GdmPKS gene cluster in *S. hygroscopicus*. Substituting the acyltransferase domain of GdmPKS module 7 (AT7) with the rapAT2 domain from rapamycin PKS [78] produced the 2-desmethyl derivative KOSN1559, which also lacks the post-PKS oxidation reactions at C-17 and C-21, as well as the desaturation at C4–C5. KOSN1559 demonstrated a 4-fold increase in binding affinity to Hsp90 compared to geldanamycin. This gain in affinity can be explained by the flexibility afforded by the saturation of 4,5-olefin, which allows KOSN1559 to more readily adopt the Hsp90-bound conformation [79]. However, due to its poor intracellular accumulation, KOSN1559 showed inferior in vitro anticancer activity compared to geldanamycin.

**Figure 19:**
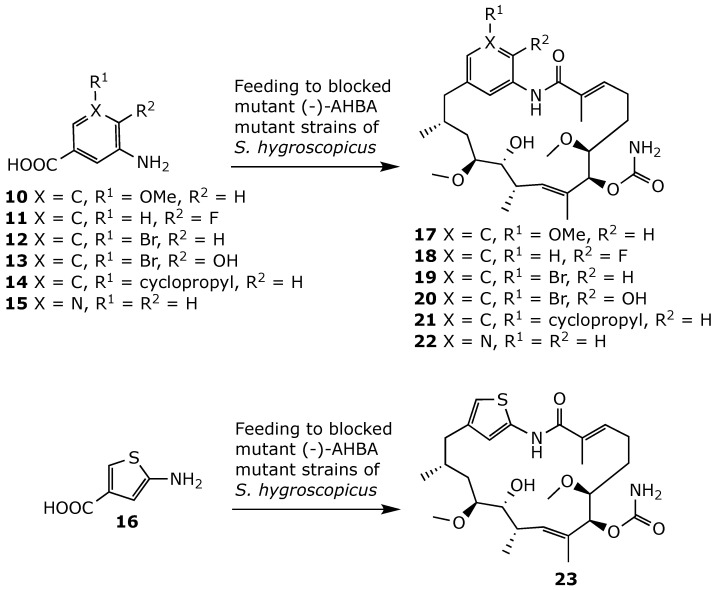
Mutasynthetic preparation of geldanamycin derivatives **17**–**23** [52].

Mutasynthesis, or mutational biosynthesis, has also been employed to develop geldanamycin derivatives in which the benzoquinone core is replaced by diversely substituted phenyl rings. Mutasynthesis involves the creation of genetic mutants of an organism that are unable to produce the key building blocks necessary for the biosynthesis of natural metabolites [80,81,82]. Supplying these organisms with structurally modified building blocks, known as mutasynthons, enables the incorporation of these precursors and the subsequent formation of novel metabolites [83,84]. In principle, mutasynthesis provides access to libraries of pharmacologically relevant and structurally complex novel compounds [52]. Thus, several groups have employed mutant strains of *S. hygroscopicus* with disruptions of the gene coding for the formation of the starter building block AHBA (Figure 2) [51,85,86,87,88]. Feeding cultures of (-)-AHBA mutant strains with unnatural 3-aminobenzoic acid derivatives **10**–**16** then resulted in the mutasynthesis of a series of novel geldanamycin analogs **17**–**23** (Figure 19). Derivatives **16**–**20** showed strong antiproliferative activity, as attested by IC_50_ values in the nM range [52,86]. By contrast, pyridyl derivative **22** of geldanamycin showed no antiproliferative activity in in vitro assays [86]. However, replacing the pyridine moiety of **22** with a thienyl ring restored the anticancer activity in **23** [52].

## 8. Targeting Specific Client Proteins

**Figure 20:**
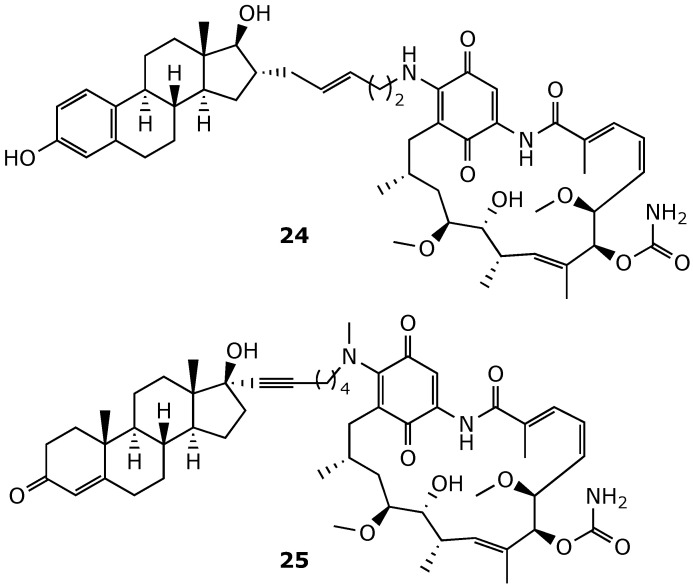
Chemical structures of estradiol–geldanamycin (**24**) and testosterone–geldanamycin (**25**) hybrids [90,91].

Since geldanamycin inhibits Hsp90, leading to the degradation of several important signaling proteins, but it lacks the ability to selectively degrade specific Hsp90 client proteins, efforts have been made to develop geldanamycin hybrids that enhance the specificity of protein knockdown [89]. One such approach led to the development of compound **24**, an estradiol–geldanamycin hybrid (Figure 20). Compound **24** demonstrated activity in the MCF7 breast cancer cell line, selectively degrading ER and HER2 while sparing Raf-1 and IGF1R [90]. Similarly, compound **25**, a testosterone–geldanamycin hybrid, exhibited selective activity against prostate cancer cell lines expressing androgen receptors [91].

## 9. Geldanamycin Prodrugs

**Figure 21:**
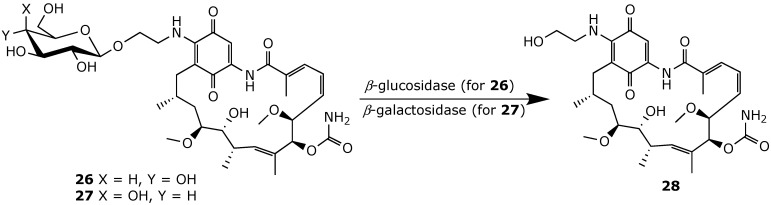
Carbohydrates-based prodrugs of geldanamycin [93].

Efforts to alleviate geldanamycin toxicity and increase its water solubility led Wang and his colleagues to employ antibody-directed enzyme prodrug therapy (ADEPT) [92]. They developed a series of geldanamycin derivatives conjugated at position 17 with a carbohydrate moiety, including glucose and galactose derivatives **26** and **27**, respectively (Figure 21) [93]. As expected, glycosylation at position 17 converted geldanamycin into inactive prodrugs, which could be enzyme specifically activated by *β*-glucosidase or *β*-galactosidase. Since β-glucosidase is ubiquitously expressed in many human cancers, glycoconjugate **26** showed potent antiproliferative activity against several cancerous cell lines, with IC_50_ values ranging from 70.2 to 380.9 nM. *β*-Glucosidase activated 26 into geldanamycin derivative 28, which was shown to have an affinity for Hsp90 similar to that of geldanamycin. However, galactosyl derivative **27** exhibited significantly reduced activity, with IC_50_ values greater than 8000 to 25000 nM. Interestingly, a 25-fold increase in the activity of **27** in the colon cancer cell line LS174T was achieved when *β*-galactosidase was selectively delivered to these cells using HuCC49∆CH2, an anti-monoclonal antibody against tumor-associated glycoprotein (TAG-72). HuCC49∆CH2 was chemically conjugated to *β*-galactosidase. This antibody–enzyme conjugate successfully targeted the tumor antigen TAG-72 while maintaining *β*-galactosidase enzymatic activity, enabling the activation of prodrug **27**. The released active drug **28** then induced up to 70% degradation of the Hsp90 client protein AKT [92]. Later, a recombinant adeno-associated virus (rAAV) was used to genetically modify animal muscles to express *β*-galactosidase. Treating these animals with prodrug 27 significantly reduced the tumor growth, compared to the control group, indicating the efficient release of the active drug by *β*-galactosidase. Additionally, serum analyses showed minimal damage to non-target organs, demonstrating that this gene-directed enzyme prodrug therapy (GDEPT) method facilitates improved localized activation of chemotherapeutic agents [94].

**Figure 22:**
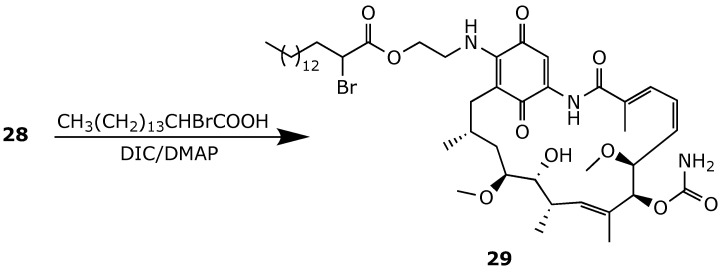
Chemical synthesis of the fatty prodrug **29** [100].

Geldanamycin derivatives that were advanced to clinical trials, such 17-AAG and 17-DMAG, are characterized by high volumes of distribution (*V*_d_) of 12.8 L/kg and 5.8 L/kg, respectively [95,96]. This high *V*_d_ limits their delivery into tumors, increases non-specific toxicities, and reduces their clinical efficacy [97,98]. To decrease the *V*_d_ and improve other pharmacokinetic properties of geldanamycins, Forrest et al. developed a series of lipophilic prodrugs of geldanamycin suitable for encapsulation in self-assembled amphiphilic block copolymer (ABC) micelles by the esterification of **28** with suitable fatty acids (Figure 22) [99]. For example, prodrug 29-encapsulated poly(ethylene glycol)-block-poly(ε-caprolactone) (PEG-b-PCL) showed a 21-fold decrease in *V*_d_, an 11-fold decrease in the total clearance (CL_tot_), and a 2000-fold enhancement in the area under the curve (AUC), compared to free 17-AAG. Interestingly, micellar **29** showed lower systemic toxicity than 17-AAG, as evidenced by a maximum tolerated dose of more than 200 mg/kg in rats [100].

**Figure 23:**
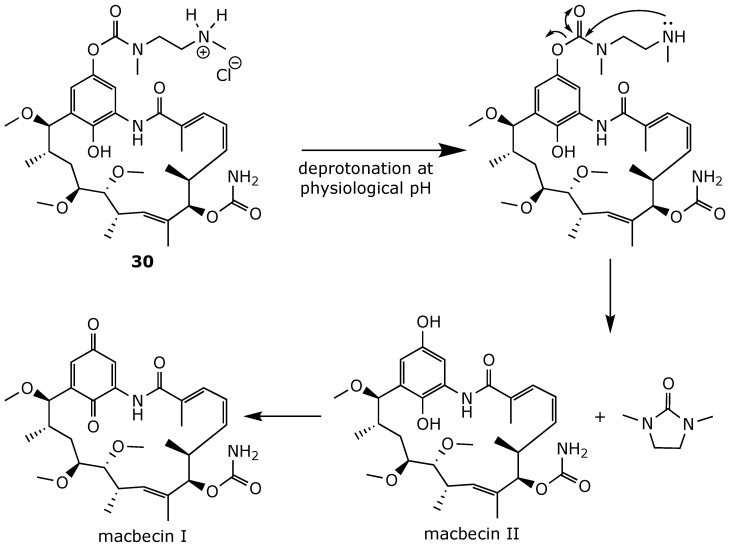
Conversion of prodrug **30** into macbecin II and then into macbecin I at physiological pH [101].

Similarly, Martin and her team developed macbecin II-based prodrugs with improved water solubility by introducing an aminoalkyl chain to the C(17)-OH position via a carbamate linkage [101]. The parent drug, macbecin II, is released at a physiological pH through an intramolecular cyclization–elimination reaction (Figure 23). In this process, the terminal amino group attacks the carbamate carbonyl, forming a cyclic urea and releasing the parent drug. For example, the hydrochloride salt 30 was cleaved into macbecin II in rabbit blood with a half-life (t_1/2_) of 49 min. Compound **30** demonstrated an aqueous solubility of more than 20 mM, 10-fold higher than that of 17-DMAG. It exhibited significant antiproliferative activity in vitro against a wide range of cancerous cell lines. Furthermore, an in vivo biological evaluation in nude mice bearing xenografts of the human prostate carcinoma cell line DU-145 showed that compound 30 significantly reduced the tumor growth rate [101].

## 10. Geldanamycin-Based Hsp90 Degraders

**Figure 24:**
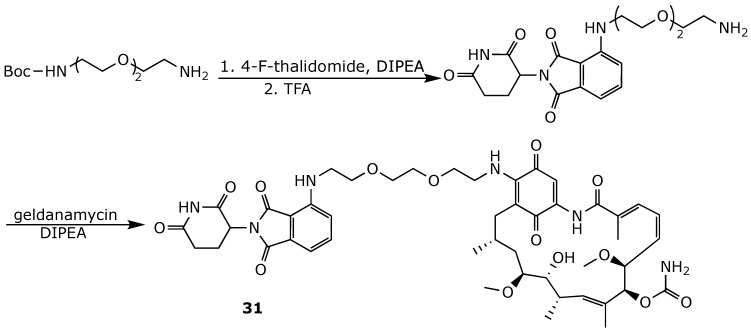
Chemical synthesis of geldanamycin-based PROTAC **31** [102].

To address the adverse effects associated with Hsp90 inhibition, Wuring et al. recently employed proteolysis-targeting chimera (PROTAC) technology aimed at degrading Hsp90 rather than inhibiting it [102]. In this approach, the cereblon-recruiting pomalidomide was introduced at position 17 of geldanamycin through a PEG2 linker (Figure 24). The so-obtained PROTAC **31** was shown to bind to the ATP binding pocket of Hsp90α-NTD with an affinity similar to that of geldanamycin, resulting in the effective degradation of Hsp90α and Hsp90β via the ubiquitin–proteasome pathway. Notably, in contrast to geldanamycin and other Hsp90 inhibitors, compound **31** downregulated the stress-inducible isoform Hsp90α in K562 leukemia cells [102].

## 11. Conclusions

Geldanamycin and its analogs have emerged as potent inhibitors of Hsp90, with high potential for use in cancer therapy. The different strategies used in developing geldanamycin derivatives significantly reduced geldanamycin toxicity. Although four of the geldanamycin analogs (17-AAG, A17-DMAG, retaspimycin, and gamitrinib) have progressed through various phases of clinical trials, none have yet fully passed clinical evaluation and gained FDA approval, primarily due to challenges such as limited efficacy, unfavorable toxicity profiles, and insufficient patient accrual [16]. These issues highlight the need for further optimization and investigation to improve their therapeutic potential.

To enhance the clinical efficacy of these Hsp90 inhibitors, strategies such as drug targeting through prodrug approaches or precision delivery systems using nanocarriers could be utilized to minimize systemic and organ toxicity. For example, conjugating geldanamycin derivatives with monoclonal antibodies targeting cancer-specific antigens could significantly enhance their efficacy while maintaining a favorable safety profile [103]. This approach would allow for more precise targeting of cancer cells, reducing off-target effects and minimizing systemic toxicity, thereby improving the overall therapeutic window of these Hsp90 inhibitors. Furthermore, targeting the cancer microenvironment by developing pH-sensitive [104,105] or hypoxia-targeted [106,107] prodrugs of geldanamycin could substantially reduce systemic toxicity. These strategies would leverage the unique conditions within the tumor, such as acidic pH [108] or low oxygen levels [109], to selectively activate the prodrugs within the tumor microenvironment. Additionally, new drug combinations can be explored to overcome therapy resistance. One potential strategy is the development of chimeric drugs that can simultaneously target multiple HSPs involved in cancer progression. Another approach is to combine immune checkpoint inhibitors with drugs that target HSP90 [16].

Overall, this review highlights the therapeutic potential of geldanamycin derivatives as anticancer drugs. Addressing the current limitations and exploring innovative approaches can significantly increase the potential of these Hsp90 inhibitors as effective therapeutic agents in cancer treatment.

## Data Availability

Not applicable.
